# The inescapable question of fairness in Pay-for-performance bonus distribution: a qualitative study of health workers’ experiences in Tanzania

**DOI:** 10.1186/s12992-016-0213-5

**Published:** 2016-11-25

**Authors:** Victor Chimhutu, Nils Gunnar Songstad, Marit Tjomsland, Mwifadhi Mrisho, Karen Marie Moland

**Affiliations:** 1 Department of Health Promotion and Development, University of Bergen, P.O Box 7807, 5020 Bergen, Norway; 2 Faculty of Social Sciences, University of Bergen, P.O Box 7802, 5020 Bergen, Norway; 3 Department of Social Science, Faculty of Education, Bergen University College, P.O Box 7030, 5020 Bergen, Norway; 4 Ifakara Health Institute, P.O Box 78373, Dar es Salaam, Tanzania; 5 Centre for International Health, University of Bergen, P.O Box 7804, 5020 Bergen, Norway; 6 Centre for Intervention Science in Maternal and Child Health, University of Bergen, P.O Box 7804, 5020 Bergen, Norway

**Keywords:** Results-based financing (RBF), Pay for Performance (P4P), Health worker motivation, Incentives, Fairness, Social relations, Distributive and procedural justice, Health sector, Tanzania

## Abstract

**Background:**

During the last decade there has been a growing concern about the lack of results in the health sectors of many low income countries. Progress has been particularly slow in maternal- and child health. Prompted by the need to accelerate progress towards these health outcomes, pay-for- performance (P4P) schemes have been initiated in a number of countries. This paper explores the perceptions and experiences of health workers with P4P bonus distribution in the health system context of rural Tanzania.

**Methods:**

This qualitative study was based on the P4P pilot in Pwani Region of Tanzania. The study took place in 11 health care facilities in Rufiji District. The study informants and participants were different cadres of health workers assigned to different outpatient and inpatient departments at the health facilities, and local administrators of the P4P bonus distribution. Thirty two in-depth interviews (IDIs) with administrators and health care workers, and six focus group discussions (FGDs with Reproductive and Child Health (RCH) staff, non-RCH staff and non-medical staff were conducted. Collected data was analyzed through qualitative content analysis.

**Results:**

The study found that the bonus distribution modality employed in the P4P programme was experienced as fundamentally unjust. The bonuses were calculated according to the centrality of the health worker position in meeting targeted indicators, drawn from the reproductive and child health (RCH) section. Both RCH staff and non-RCH perceived the P4P bonus as unfair. Non-RCH objected to getting less bonus than RCH staff, and RCH staff running the targeted RCH services, objected to not getting more P4P bonus. Non-RCH staff and health administrators suggested a flat-rate across board as the fairest way of distributing P4P bonuses. The perceived unfairness affected work motivation, undermined teamwork across departments and created tensions in the social relations at health facilities.

**Conclusion:**

Our results suggest that the experience of unfairness in the way bonuses are distributed and administered at the health facility level undermines the legitimacy of the P4P scheme. More importantly, long term tensions and conflicts at the workplace may impact negatively on the quality of care which P4P was intended to improve. We argue that fairness is a critical factor to the success of a P4P scheme and that particular attention should be paid to aspects of workplace justice in the design of P4P bonus structures.

## Background

Many low-income countries did not meet the targets set by the Millennium Development Goals (MDGs) 4 and 5 to improve child and maternal health [1]. During the last decade there has been a growing concern about the lack of positive results in the health sectors of many low income countries, in particular in maternal health, and new strategies to improve these health outcomes have been introduced. One such strategy is pay-for-performance (P4P) which is embraced by many policy-makers as a way to achieve progress towards these goals [2]. P4P is defined as “transfer of money or material goods conditional on taking a measurable action or achieving a predetermined performance target”[3]. With its focus on results, P4P fitted into the MDG paradigm, where attention was given to a few targeted indicators for rapid progress [2]. To date, over 30 low-income countries (LICs) are currently implementing P4P projects, with the majority of them in sub-Saharan Africa [4].

Limited research has been carried out on the effectiveness of P4P in LICs and it is not known whether P4P will facilitate health system improvements in the long run. Two evaluation studies assessing the effects of P4P in Rwanda and Tanzania concluded that the schemes had led to an increase in the usage of certain maternal and child health services, including facility deliveries [5, 6]. The Rwanda study, a randomized controlled trial, found that P4P incentives had the greatest effect on the services that had the highest bonus payment rates and needed the least efforts from the service provider, i.e. the health worker [5]. The study was able to isolate the incentive effects from resources effects by increasing control facilities’ input-based budgets by the average P4P payments made to the intervention facilities. Hence is often cited as one of the best examples on measuring the effectiveness of P4P [7]. However, in Rwanda another study found that P4P encouraged the prioritization of rewarded services and a neglect of unrewarded services [8]. These behaviors are called *gaming* in the P4P literature and can be detrimental to the quality of health care [9].

Two systematic reviews on the effectiveness of P4P schemes in low to middle income countries concluded that such schemes are less likely to create sustained changes in health service delivery [3] and that there is weak evidence to support that these schemes work [10]. The fact that available evidence is conflictual, fuels the debate on whether to use P4P schemes in the health sectors of low income countries. While this debate goes on P4P schemes are increasing exponentially in the sub-Saharan African region, which contributes to the importance of generating new knowledge on *how* some specific components of the existing P4P schemes work and are perceived in local social settings. It has been noted that social-cultural aspects plays an important role as to *why* the success of P4P schemes varies across contexts [11], and *how* fairness principles are perceived [12, 13]. Yet to our knowledge, no research to date has attempted to address *how* P4P bonus system, an important feature of the scheme, is experienced in the social-cultural settings of low-income countries. Our study attempts to bridge this gap by exploring how health workers in local health facilities perceive and experience the P4P bonus structure, paying particular attention on *how* fairness is perceived and *how* this may affect social relations at local health facilities in Tanzania.

### Theoretical framework

P4P rests on some fundamental principles and concepts that are important to clarify. A commonly used definition of motivation in the work context is “an individual’s degree of willingness to exert and maintain an effort towards organizational goals” [14]. Behind this definition lies an assumption that effort and work towards the organizational goals can be improved. In an employer-employee relationship the employer wants to measure and if necessary adjust the performance of the employees. An influential approach to workplace motivation is formulated through the principal-agent theory in economics [15]. The principal-agent theory asserts that there often is lack of correspondence between the interests of the principal (employer) and the agent (employee) when it comes to the goals to be achieved by an organisation. To overcome this, the principal tries to find ways of aligning the agent’s goals with the goals of the organisation. P4P can be seen from this perspective. The implication of the principal-agent theory is that increased alignment between the goals of the principal (employer) and the agent (employee) can be achieved in a number of ways including the offering of financial incentives.

Motivation is fundamental for both the quality and quantity of the work performed. Motivation theory commonly distinguishes between intrinsic and extrinsic motivation [16]. Ryan and Deci postulates that “[t]o be motivated means to be moved to do something. Thus a person who feels no impetus or inspiration to act is characterized as unmotivated, whereas someone who is energized or activated toward an end is considered motivated” [16]. In the health sector, both intrinsic and extrinsic factors of motivation are considered important. Ryan and Deci defines intrinsic motivation as “doing something because it is inherently interesting or enjoyable” and extrinsic motivation as “doing something because it leads to a separable outcome” [16]. Extrinsic factors for motivation encompass diverse incentives and mechanisms expected to encourage a worker to increase the effort to perform workplace tasks. The balance between intrinsic and extrinsic motivation varies between employees and over time. These two types of motivation may influence each other. For example, increased extrinsic motivation through availability of incentives may diminish intrinsic motivation for the workplace tasks [17]. P4P offers financial incentives to health workers upon reaching a predefined threshold in performance on certain indicators. Generally, only selected groups of health workers are entitled to receive such financial incentives, in this case health workers working with child and maternal health. The difference in access to such incentives within health care facility may impact individual perceptions of fairness and justice in the P4P scheme. An important distinction in studying these perceptions is between distributive and procedural justice. These two notions of justice have traditionally been treated distinctly in the literature on the psychology of justice.

Distributive justice concerns allocation of goods, for example salary and financial incentives, whereas procedural justice concerns “perceptions of the fairness of decision-making processes” [18]. Given the importance of these notions at the workplace, there have been attempts to link these two notions in a single framework through *the referent cognitions theory* [18, 19]. Folger argues that *the referent cognitions theory* makes the relationship between these two notions explicit, highlighting on the basis for the effects that procedures have on perceptions of distributive justice [18]. The theory postulates that when an outcome is seen as unjust or unfair, the result is not merely dissatisfaction but a sense of moral outrage which cause resentment. Therefore, the experience of resentment towards an unfair outcome can be analyzed in two ways, that is, consideration of an imaginable better outcome that could have been more satisfying (distributive justice), and improper procedures or processes that may have hindered the attainment of the desired outcome (procedural justice). An underlying basic premise for the theory is that employees of any organization tend to prefer fair treatment over unfair treatment, based on their perceptions on what is fair. Thus employers have a clear stake in fairness as “they would not expect to find their most dedicated and loyal employees among those who feel unfairly treated” [18].

Therefore, while the principal-agent theory promotes the use of financial incentives, if not used in a proper way such incentives may impact on perceptions of distributive and procedural justice at the workplace. Thus, a P4P scheme can be assessed both in terms of the actual distribution of incentives, and in terms of the principles, processes and circumstances behind the distribution. In the implementation of P4P schemes in the health sector, the following questions are rarely asked, although they actually are quite fundamental: 1) how does P4P incentive distribution modalities impact on perceptions of fairness and health workers’ motivation, and 2) what is the impact of the P4P incentive distribution on relations among health workers at health facilities.

Drawing on narrative interviews with health workers who have experienced working within a P4P scheme in Tanzania, this article attempts to provide some answers to these questions.

### Pay-for-performance (P4P) in Tanzania

Tanzania is one of the African low income countries that have embarked on a strategy to implement P4P in the health sector. The efforts towards meeting MDG 4 and 5 in Tanzania were followed by a significant reduction in child mortality (in 1996: under five mortality was at 137 deaths per 1000 live births, and in 2010 it had declined to 81 deaths per 1000 live births), while less improvement has been achieved in maternal health (in 1996: maternal mortality was 529 per 100,000 live births, and in 2010 it was at 454 per 100, 000 live births) [20, 21]. Following the public debate on how to meet the health related MDGs by 2015, the first plans to introduce a national P4P scheme were launched in Tanzania in 2007. The Government of Tanzania wanted a nationwide roll-out of P4P and argued that “P4P constitutes an important programme strategy which will increase the ability to unleash the energy and creativity needed to address local challenges” [22]. The international donor community was hesitant about the feasibility of the planned P4P [23], but despite these concerns, the Government of Tanzania went ahead implementing P4P in a few districts [24, 25]. In response, the international donor community pulled out the funding of this P4P programme [24]. Consequently, the P4P programme of 2009 ended up having very limited success.

After a series of discussions and consultations between the Government of Tanzania and the international donor community, a more comprehensive plan for a P4P scheme was developed with greater focus on relevant indicators and on the need to strengthen the Health Management Information System (HMIS) [26]. The result was a P4P pilot in 2011 that was implemented in the Pwani Region, whose evaluation has been completed [6, 27]. The P4P pilot in Tanzania received technical support from Clinton Health Access Initiative (CHAI) and financial support from Norway [26].

Indicators for the P4P pilot in Tanzania are drawn from the reproductive and child health (RCH) department, with the majority coming from maternal and child health section in line with the need to accelerate progress towards unmet Millennium Development Goals 4 and 5. The indicators cover the following areas: antenatal care, institutional deliveries, post-natal care, prevention of mother-to-child transmission of HIV (PMTCT), family planning and health management information systems [26]. The process leading up to P4P in Tanzania has been reported in one study as by and large a top-down process which did not include the views of important stakeholders such as, frontline health workers [28] who are strategically positioned between patients and administration.

Guidelines for payment of the incentives are outlined in the Pilot Design Document [26]. Incentive payments are provided through the health facility and the funds are divided into two parts; one for staff incentives and one for facility improvements. For hospitals, 10% of the incentive payment goes to facility improvements, while 60% is awarded reproductive and child health (RCH) staff, and 30% non-RCH and non-medical staff. For dispensaries and health centres, 25% goes to facility improvements while 75 % goes to staff bonuses. The guidelines recommend that bonus payments are shared equally among RCH and non-RCH staff at dispensary and health centre level, but this is practiced differently. In some dispensaries and health centres RCH staff and non-RCH staff received different bonuses. The average bonus payment per month for most health workers is approximately USD 30, which is about 10 percent of the average monthly salary of nursing staff [26]. However, for non-RCH and/or non-medical staff at hospitals the figure can be significantly lower [26]. The bonuses are paid twice a year, following a 6 months P4P pilot cycle. The rules for payment are that if less than 75% of the target is achieved, the payment for that indicator is 0, if 75–99% of the bonus is achieved, 50% of the bonus on that indicator is paid, and if 100% percent is achieved, then a full bonus is paid on that indicator [26].

The total amount allocated for the P4P pilot per cycle is USD 300,000 and assuming that the pilot is scaled-up across all regions in Tanzania, P4P will require approximately USD 16 million per year [26]. The design document outlines maximum potential bonus payments per health facility taking into account the typical staff profile of each facility type. Hence, hospitals can have a potential maximum P4P bonus of approximately USD 8000 while health centres can have up to USD 2800, and dispensaries a potential maximum bonus of USD 700 per cycle of 6 months [26]. Under the P4P scheme, incentives are financial and the P4P design document does not specify any non-financial incentives[26].

## Methods

### Study setting

The study was carried out in Rufiji District, one of the six districts in the Pwani Region which is piloting P4P in Tanzania. Rufiji is a rural district and according to the 2012 national census, it had a population of 217,274 [29]. Administratively, the district is divided into 27 wards, 96 villages and 400 hamlets [29, 30]. It has a total number of 64 health facilities, including two hospitals, five health centres, and 57 dispensaries [30]. Like many rural districts in Tanzania, Rufiji district faces significant shortages of staff and of the 583 positions in the district health sector, only 301 are filled [30].

The main feature of the district is the Rufiji River which divides it into approximately equal geographical halves. The majority of the district’s population lives in the northern part, which is easily accessible throughout the year. The southern part of the district as well as the river delta zone may be inaccessible for varying periods of time due to long and heavy periods of rain. The only means of transport to the delta zone are canoes and boats. As a result, for long periods every year health facilities in this zone face huge problems in procuring medical supplies and in communicating with the district health offices located at Utete, the district capital situated in the southern part of the district. This area also finds it difficult to attract qualified health workers and the district lags behind in health outcomes compared to other districts in Pwani Region. However, in comparison to national averages the district is doing well, for example the district’s maternal mortality rate was at 306 compared to the national average of 454 per 100,000 live births [21, 30].

### Study design and data collection

A qualitative study approach was adopted to explore the perceptions and experiences of health workers with P4P. Data was collected by the first author together with a research assistant during a 6 months period between January and June 2013. Purposive sampling was used for the selection of health facilities and recruitment of informants. In-depth interviews (IDIs) and focus group discussions (FGDs) were used as main data collection methods.

Data was collected at 11 health facilities, with the aim to cover all levels in the health referral system. In addition, we aimed at covering a wide geographical distribution of health facilities, and the furthest facility in our sample is approximately 50 km from Utete. Due to poor accessibility we did not include health facilities in the delta zone. In our sample we had two hospitals, two health centres and seven dispensaries. Of these 11 health facilities, four were church run and the remaining seven were publicly owned.

### Research participants

Research participants were recruited to cover diverse categories of health workers at health facilities. Additionally, at all health facilities in our sample we interviewed the in-charge or their substitute. The rationale for these selections was to get an assessment of how P4P functioned at the particular health facility as seen from different perspectives including that of the leadership and of the different cadres of health workers. It was important to include health workers from different wards and departments of the facility since the P4P programme targeted these differently. A total of 32 IDIs with health workers of different cadres ranging from medical officers, assistant medical officers, clinical officers, nursing staff, laboratory staff, medical attendants and an official from the district health office were conducted, as summarized in Table 1. An interview guide was used covering the following themes: perceptions on individual work motivation, work relations, and experiences with P4P bonus distribution.

**Table 1:** Overview of IDIs

Category of informant	Number of interviews
Medical Officers (MO)	2
Assistant Medical Officers (AMO)	2
Clinical Officers (CO)	3
Nursing staff	11
Medical attendants (MA)	11
Laboratory staff	2
Official from the district health office	1

In the course of the research, we learnt that when it comes to the issues of bonus distribution, health workers held collective views, depending on whether they were RCH or non-RCH staff (including non-medical) and also depending on how a particular facility was distributing the bonuses. To pursue these collective views we conducted FGDs with health workers from different wards. Six FGDs with a total number of 30 participants were conducted, three different work-area categories: three with RCH staff, two with non-RCH staff and one with non-medical staff, as summarized in Table 2.

**Table 2:** Overview of FGDs

FGD number	Category of staff	Location	Participants	Men	Women
FGD 1	RCH	Hospital K	5	1	4
FGD 2	RCH	Health Centre D	5	1	4
FGD 3	RCH	Dispensary B	4	0	4
FGD 4	non-RCH	Hospital K	6	3	3
FGD 5	non- RCH	Health Centre E	5	3	2
FGD 6	non- medical	Hospital H	5	4	1

### Data analysis

All interviews and FGDs were conducted in Swahili, except two interviews with medical officers which were conducted in English. The first author speaks colloquial Swahili, while the research assistant was a Tanzanian citizen with experience in qualitative health services research. All interviews and FGDs were recorded, transcribed and translated. In addition, rapid note taking was used. Verbatim translations from Swahili to English were error checked. Qualitative content analysis was used as the mode of analysis [31] and the transcripts were subjected to a thorough review before the coding exercise began. After coding, meaning units were condensed and interpreted and from these, emerging sub-themes and themes were identified. Refer to Table 3 for an overview of the analytical process from codes to themes. OpenCode 3.6, a free software for qualitative data analysis [32] was used for data management.

**Table 3:** Examples of meaning units, condensed meaning units, sub-themes and themes in qualitative content analysis

Category: bonus distribution concerns in OpenCode 3.6				
Meaning unit	Condensed meaning unit	Interpretation underlying meaning	Sub-theme	Theme
	Description close to the text			
The report for RBF comes from RCH every end of the month, but when the bonus come it is shared equally, even with ecurity guards	RCH staff do more work	RBF indicators are from RCH	Negative perceptions towards RBF bonus distribution and their influence on social and work relations	Perceived unfairness over RBF bonus
I think it is a normal problem for human to fight for money. Sometimes you see someone who doesn’t even put an effort at work claiming that they need more bonus share	Money always cause problems	Sharing money is a problem		
RCH’s work is important but they cannot accomplish this task alone, why then do they need more bonus than anyone else. Everyone deserves the same RBF bonus.	Flat rates are fair ‘everyone toils’	A flat rate need to be used for RBF bonuses		
When we sit and try to solve our problems here concerning RBF bonus, the RCH staff do not support me because RBF favors them. I see this program has some negative impacts. Just imagine you have a family and you give food to one of your child while the others are looking	RBF encouraging conflicts among workers	RBF bonus distribution causing conflicts		
We normally get our bonuses too late. Sometimes, some people can get their money early while others get it late and we wonder how this is possible	The need for RBF bonus to be distributed timeously	Bonus is delayed	RBF management concerns/problems	
Sometimes we do good work and report good data, but during the verification process, somehow we always end up with lower figures. This affects our RBF bonus money. We don’t know what they take into consideration	Data is not captured properly	RBF data is not captured properly		
The thing I don’t like about RBF is that it doesn’t consider the workload. As you can see we are a dispensary here but we do a lot and serve many people, …sometimes more than a health centre but when RBF bonus money come they don’t consider that workload or the number of people we serve	Target setting not fair	The criteria for setting RBF targets not fair		

## Results

A major topic in the IDIs and the FGDs that will be examined in the Results section concerns fairness in the distribution of P4P bonuses. The following presentation of results is divided in two parts that are both thematically framed within the broad theme of fairness.

The first part presents the health workers’ perceptions of the influence of the P4P bonus modality on interpersonal, inter-departmental and work relations. This section focuses in particular on the different positions of two groups, the RCH and non-RCH staff (including non-medical staff in the case of hospitals), and the implications that the different positions had on work relations. The second part presents opinions about P4P target setting and the day-to-day management of the pilot, with particular attention to the criteria used in determining maximum potential facility bonus, data verification procedures and bonus payment delays.

### Interpersonal and inter-departmental relations

#### We do a lot here in RCH

The large majority of the informants working in the RCH departments maintained that the current P4P bonus distribution modality at dispensaries and health centres, where they, unlike at hospitals get equal bonus money with non-RCH staff was fundamentally unfair. This perception stemmed from the fact that most P4P targets are related to tasks in the RCH department. RCH staff felt that they deserved higher bonuses than their colleagues in other wards of the health facilities. As one informant put it:

*“You see P4P indicators are from RCH, we do a lot here to meet P4P targets, a person from the laboratory doesn’t even touch the P4P report, and even our doctor here doesn’t know how to fill that report. Yes, we work with them but it is only us who prepare the P4P report and because of this RCH need to get more bonus money. You know during the first round we even got the same amount as security guards, how is this possible?”* (Nurse-RCH staff, Dispensary A, IDI)

The P4P Design Document allows flexibility on how to distribute P4P bonuses. This, however, created much variation in the distribution of bonuses, even among health facilities at the same level in the national health system. Some dispensaries and health centres decided to give RCH staff more bonuses than non-RCH staff and this was equally perceived as unfair by non-RCH staff, as the following quote shows:

*“When P4P came we thought it was for all. But later we discovered it wasn’t even for me, a doctor or other staff, it is only for RCH. We don’t know why it is for some people only while we all work here. How come only some are benefitting? We were all happy in the beginning, but we came to know that there was a special group chosen. During the last distribution, I was given as little as TZS 18,000,* (RCH staff at this facility got TZS 120,000) *just the same as a security guard and a laundry man”* (Assistant medical officer- non-RCH staff, Dispensary B, IDI)

#### P4P bonus distribution discourages us

There was deep frustration among non-RCH staff that was getting less P4P bonuses than RCH staff. As P4P targets mainly the RCH department, health staff working in other areas, like the out-patient department, may now put less effort into their work than before due to the fundamental feeling of being unfairly treated:

*“When the program started we were motivating each other to work hard and together. But later we heard that some staff will not be paid and we became discouraged. There are a lot of things we do to assist the RCH department, like encouraging pregnant mothers and or encouraging them to come with their children for check-ups, including even treating them. But when the P4P bonus started to create these groups we became automatically discouraged because even if you make an effort, you will not be paid as others. Why work in order for others to get money?”* (Non-RCH staff, Health Centre E, FGD 5)

While the distribution key used varies in health centres and dispensaries, in hospitals, RCH staff always gets more bonus payments than non-RCH and non-medical staff. In our data, we found the reported cases of workers demanding to work in the RCH department in order to get higher P4P bonuses. While this in many ways is a positive development, especially for the maternal and child health section which is traditionally perceived to be dirty and demanding, there is a need to ensure that all sections at a health facility are adequately and fairly staffed. One medical doctor noted this problem:

*“The problem is that an element of selfishness is present when people think of money, especially when a person works in a section getting less P4P bonus knowing that someone is getting more money in another section. In the end you find that everyone wants to work in one section, the RCH”* (Medical officer-non-RCH staff, Hospital H, IDI)

In hospitals, there are three different groups when it comes to the distribution of P4P bonuses; the RCH, non-RCH and non-medical staff. These different categories get different amounts of bonuses, with the RCH getting most and non-medical staff least. For example, at one hospital RCH staff received TZS 107,000, while non-RCH staff got TZS 85,000 and non-medical staff got TZS 65,000. Non-medical staff felt less appreciated as they argued that they are an important component of the health system. They saw P4P as the only kind of incentive available to them, while other health workers also have opportunities for other types of allowances:

*“The program is good, but the problem starts when the money is distributed. My opinion is that the money should be shared equally to all because all workers have their own responsibilities. The RCH does more on P4P records but we have to keep in mind that it is their duty. For example a driver does a lot, taking RCH people to outreach and assisting them with their work. P4P is the only motivation we have; other high ranked staff can go for trainings and seminars* [where they have opportunities to get per diems], *what about a security guard?”* (Non-medical staff, Hospital H, FGD 6)

#### P4P is causing segregation and tensions

Frustrations with the distribution modality of P4P bonuses under the pilot were evident across cadres and facility types. One group or another always felt unfairly treated, independent of which distribution modality practiced by the health facility in question. The data indicated that P4P was causing tension among workers in different sections at health facilities. These frustrations came from the RCH staff who felt they deserve even more bonuses, as well as and from non-RCH or non-medical staff who felt that they were being unfairly treated by getting lower P4P bonuses. For staff in supervisory roles, P4P had created an uneasy working situation:

*“P4P segregates other workers and to me that is the problem. For example, the security guards are not involved in treatments but they are an important part of the dispensary. As a supervisor of the dispensary I see them all as important in meeting our objectives. If a security guard doesn’t perform his duty well and some damage or theft happen at the RCH, how will they deliver their patients? Is it fair to give him less P4P money? Can you give food to one of your child while the others are just looking?”* (Clinical officer-non-RCH staff, Dispensary A, IDI)

#### A flat rate is better

In both in-depth interviews and focus group discussions P4P bonuses were perceived to create an uneasy work environment at health facilities. Some of the informants suggested that flat rates across cadres would be the fairest way to distribute the bonuses; however, this option was mostly preferred by non-RCH staff, non-medical staff and those in supervisory roles at health facilities. The following quote illustrates this point:

*“This P4P money to us is just like an* ***asante*** (thank you). *You can’t divide an* ***asante*** *and say this one will get a bigger portion because they have a higher training or because they do more work. Every worker here has a salary and we know and agree that a salary need to consider the level of education and training but not an* ***asante*** *. However, people are selfish when it comes to money, you start to hear many staff categories and this destroys the motivation they intend to build. Personally I think a flat rate is fair.”* (Non-RCH, Hospital K, FGD 4)

The non-RCH staff in Rufiji argued that a flat rate is fair. They were aware that RCH staff has extra duties when it comes to P4P targets. However they considered P4P bonuses as a ‘gift’ which should not be differentiated by tasks or level of training. Workers in different wards expressed willingness to have P4P targets related to their own duties. When we asked a representative from the district health offices in Rufiji if bonus distribution was an issue of concern, we got the following response:

*“Bonus distribution is a problem, in one way the RCH plays a big role and P4P’s main focus is to reduce maternal- and infant mortality. On the other hand, non-RCH also plays an important role in this since RCH staff cannot do their tasks independently of others. When it comes to payment we are facing this problem and non-RCH a staff is threatening to leave all the tasks related to P4P bonuses to the RCH staff. If this happens, the RCH staff cannot manage these tasks alone. Therefore my opinion is to make the rates flat so that everyone can participate effectively knowing that we are going to get the same bonuses. This also helps in reducing the tensions we face since the introduction of P4P.* (Official from district health offices, Utete, IDI)

### P4P management and target setting concerns

The section present three issues: the criteria used by the pilot in determining maximum potential facility bonuses, data verification procedures and bonus payment delays.

Maximum potential bonuses for facilities are determined by facility type. For example, dispensaries have a similar potential maximum bonus. However, in practice facilities at the same level of the referral system possess a number of variations, such as in the number of people they serve and the number of staff available. Some dispensaries in the study area had as few as two health workers, while others had as much as twelve, way above the nominal staff establishment at dispensaries which is five. Health workers in the dispensaries with more staff and a big catchment area preferred that the criterion for maximum potential bonus be based on a case-by-case analysis, as the following quote shows:

*“The thing I don’t like about P4P is that it doesn’t consider the workload. As you can see we are a dispensary here but we do a lot and serve many people. For example our vaccination target is of 60 children per month, and yet other dispensaries can have a target of as little as five but in the end we are all paid an equal P4P bonus. And let’s say if we don’t reach 40 children, which is 80% of the target, we get nothing despite the hard work. Yet someone with five children, which is their 100%, are paid fully. So you see. It is not ok.”* (Nurse- RCH staff, Dispensary A, IDI)

The assumption behind the criterion in the current P4P design is that facilities with big catchment areas possess a great potential of reaching their P4P targets, hence the need for these facilities to have higher targets than those with a smaller catchment area. Regardless, health workers perceived this as contributing to the fundamental feeling of unfairness that they all seemed to share.

The verification of data was also noted by our informants as problematic. Data verification in the Pwani pilot is done by the Clinton Health Access Initiative (CHAI), which manages the pilot on behalf of the Ministry of Health and Social Welfare. Health workers complained that there were a number of disparities that arose from the data they reported and the bonus payment they received as a facility. Health workers explained that the figures they had reported were considered by data verifiers to be inflated. These claims were widespread, regardless of facility type. The following citation captures this concern:

*“Sometimes we do good work and report good data, but during the verification process, somehow we always end up with lower figures. This affects our P4P bonus money. We don’t know what they take into consideration.”* (RCH staff, Dispensary B, FGD 3)

It could be that health workers are still learning to properly capture data, or that the verification team misses some important data during verification, which is costly for health facilities.

Delays in bonus payments were also noted as a major concern. Some of the payments were delayed by as much as a full P4P cycle [6 months]. A number of our informants expressed this concern:

*“We normally get our bonuses too late. We have had a situation where people nearly fought in order to get their bonuses. At this facility, sometimes, some people can get their money early while others get it late and we wonder how this is possible. We need to get the money in our bank accounts just like our salaries”.* (Non-RCH staff, Health Centre E, FGD 5)

The delay in bonus disbursement was a problem which was also noted by the district health management of Rufiji District. As shown by the following quote:

*“Generally we think P4P is a good thing, but of course there are concerns like that bonuses have to be increased or that they are delayed or concerning the distribution modalities at facilities. On the delays we have to admit that it is also our internal problems and we are partially responsible for the delays.”* (Official from district health office, Utete, IDI).

Delays in bonus disbursements were attributed to a number of reasons, including delays in endorsements by the district management, which was a requirement before representatives of the respective health facilities could withdraw their P4P bonuses from the bank. In addition, some church run and some private health facilities had no bank accounts, which was a requirement to get bonuses. In such cases, the money had to pass through the bank account of someone in the district leadership, which potentially causes more delays in payment of P4P bonuses.

## Discussion

Perceptions of injustice in the P4P bonus distribution among recipients are dominant in our findings. To shed further light on these findings, two questions warrants discussion: Why did health workers experience the P4P bonus structure as unfair? And what are the implications of the perceived injustice on motivation, teamwork and social relations among health workers in particular and to the health sector in general?

### P4P bonus structure: inescapably unfair?

Our data indicates a fundamental feeling of unfairness relating to both actual financial incentive payouts and the process and criteria behind the rewarding process. We will use *the referent cognition theory* [18] and its two notions of procedural and distributive justice at the workplace to elucidate the link between the P4P bonus structure and health workers’ feeling of unfairness.

Our data presents a clear divide between two groups of health workers, that is, RCH staff and non-RCH staff (including non-medical staff at hospitals). These two groups had three different stakes when it comes to P4P incentive payouts. The RCH staff at hospitals got more than non-RCH staff and argued that they deserved it. The RCH staff at dispensaries and health centres got an equal bonus amount as non-RCH and argued that they deserved more. Finally the non-RCH staff at hospitals got less bonus payouts than the RCH staff and argued that they deserved more. In other words, one group, the RCH staff at hospitals, was satisfied with the outcome (bonus payouts) while the other two groups, the non-RCH staff at hospitals and RCH staff at dispensaries and health centres, were unsatisfied. These perceptions relates to the notion of distributive justice. The *referent cognitions theory* predicts that people (workers) may be more resentful to an undesired outcome for two reasons: i) if they imagine a better outcome could be obtained instead and, ii) if the events, actions, or circumstances that prevented the better outcome seem to be improper [18].

The two groups of health workers unsatisfied with their bonus payouts, that is, the non-RCH staff at hospitals and RCH staff at dispensaries and health centres, had different perceptions on why they deserved better payouts. The RCH staff at dispensary and health centres argued that they are contributing to the P4P indicators directly; hence they should get a different rate than their non-RCH colleagues. This argument is supported by the logic of the principal-agent theory. The theory promotes the alignment of individual and organizational goals, and P4P is one mechanism aiming to achieve that by emphasizing the contingency of results and rewards. This supports the claim that RCH staff in dispensaries and health centres working directly to improve the health outcomes targeted by P4P deserved a higher rate of bonus payouts as their counterparts in hospitals. Thus when comparing themselves with RCH staff in hospitals, the RCH staff in dispensaries and health centres perceived the outcome to be too small and the bonus structure to be distributively unjust.

Non-RCH staff viewed the bonus structure as both distributively and procedurally unjust. They argued that they deserved more from the effort they put into their work, which they claimed to be no less than the RCH staff. Therefore, according to the notion of distributive justice, they resented the current outcome (bonus payouts), because they anticipated a better outcome [18]. Unlike the RCH staff at dispensaries and health centres, non-RCH staff’s claim for more payouts is mainly based on procedural justice. In our study context the aim of P4P was to accelerate progress towards MDG 4 and 5, and in this regard the RCH staff became central.

However, considering that P4P was a top-down reform necessitated by unmet health care needs, one can argue that the circumstances and processes leading to the adoption of P4P, as a reform, had little to do with local priorities and perceived health system challenges in Tanzania. The design of P4P, its priority areas and bonus distribution modality was top-down, driven primarily by an international agenda [28]. Hence non-RCH staff contends that P4P was a result of an improper process, a process out of their control and yet they were sanctioned for factors and decisions out of their control. Non-RCH-staff claimed that they were deprived of the opportunity to earn as much as their colleagues working with RCH-services simply because P4P indicators were selected from RCH services.

Our data points to an important discussion relating to the issue of fairness in P4P scheme. An early critic of P4P, Berwick claims that the P4P mechanism is toxic in the health sector and fraught with contested values [33]. The current modality which pays according to the effort towards targeted health services is contested and so is the alternative modality of a flat rate. One study carried out in Tanzania on the P4P scheme that was partially implemented between 2009 and 2011, documented that one district, Mvomero, decided to use a flat rate, citing fairness and equity concerns, among other issues [25]. The same concerns were highlighted by those in supervisory roles at health facilities in this study. Fairness is an important aspect to consider in many socio-cultural settings, and the socialist recent history based on the philosophy of *Ujamaa* (African communal living) [34, 35], may further increase the emphasis put on this in Tanzania. Through nostalgia, some Tanzanians, including health workers still perceive the state as a fair and equitable distributor of goods and services [36, 37]. Therefore, the unequal P4P bonus distribution goes against the grain of this thinking in this particular socio-cultural context.

Furthermore, our study indicates the importance of structural and management concerns in a P4P scheme in order to avoid reproducing procedural injustice. Our study highlighted that perceived structural and management anomalies in a P4P scheme contributed significantly to the fundamental feeling of unfairness. For example, informants in our study reported a number of irregularities with data verification, facility classification concerns and delays in bonus payouts. According to *the referent cognitions theory* [18], when people (workers) perceive the circumstances that prevented the better outcome (more bonus payout) as improper, their resentment for desired outcome increases.

Our data has shown clearly that the bonus structure of the Pwani P4P pilot, which uses more or less the same modality as other P4P schemes being implemented in large parts of sub-Saharan Africa, is prone to create and reproduce feelings of injustice at the workplace. This finding is supported by Berwick’s claim that P4P is inescapably unfair [33], hence there is reason to believe that this finding is relevant and valid beyond the study setting. This feeling of unfairness at the work place has implications for workers’ motivation, performance and fundamentally social relations. Magrath and Nichter called for P4P schemes to pay particular attention to social relations in different socio-cultural settings, in the following section, we heed to this call using our study as a case [11].

### Unfairness: implications on motivation, teamwork and social relations

The feeling of injustice related to P4P has unintended effects on motivation, teamwork and social relations at the workplace [11] which all affect performance and the quality of care. Motivation in general is important at the workplace, and intrinsic motivation is of great importance for health workers in providing quality health services [38–40]. While there is no conclusive evidence on the effects of P4P on intrinsic motivation in the health sector, studies from other sectors, including that of Deci and Ryan have concluded that the introduction of external rewards undermines the intrinsic interest of performing a task [17, 33, 41]. Studies in Rwanda have highlighted both the potential benefits of P4P [5], and the potential unintended effects of P4P [8, 42]. What comes out clearly in these studies is that P4P affects the motivation of health workers in a number of ways. Given the effect on motivation, which in the context of our study can be argued to be negative, we will now move on to discuss how this affects teamwork and social relations at the health facility.

Teamwork is a salient feature in health care. A number of studies have highlighted the importance of teamwork in health care in Tanzania and other contexts [43]. P4P bonus distribution seems to trigger divisions among health workers based on the classification and eligibility to P4P bonus pay-outs. Health workers are becoming more conscious of the staff categories RCH and non-RCH (and non-medical staff) than before the introduction of P4P. This new emerging consciousness and identities at health facilities create barriers for teamwork, which health care work relies on.

In our study, non-RCH staff mainly in hospitals is becoming reluctant to help the RCH department, the reason being that the RCH are the major beneficiaries of P4P bonuses. This, it can be argued, has serious implications to the overall quality of services offered to patients. The need for teamwork in health services in resource constrained settings, for example Tanzania, cannot be overemphasised [44]. In our study a number of low-level staff reported to substantially help qualified staff, but these workers are normally classified as non-medical staff which has implications for their share of the P4P bonus distribution. The reduced motivation and reduced teamwork may have had an effect on the social relations among health workers.

The use of incentives and sanctions can potentially turn working relationships into a commercial contract [33] and may have negative consequences for the working environment and the social relations at the workplace. The collectively shared values that nurture relations, creativity and ingenuity at work are outside the realm of P4P. Establishing trust for instance is foundational to health care, but such process indicators are not measured in P4P and hence not eligible to bonus pay-outs. With an incentive structure perceived as unfair and that promotes outcomes and not processes, trust instilled in patients through respectful interaction may be undermined by time pressure and dwindling motivation.

## Limitations

This study aimed to explore the perceptions and experiences linked to P4P bonus distribution modality and hence, did not address wider experiences and perception with P4P. It was conducted in only one district out of six in the Pwani Region implementing the P4P pilot. The general characteristics and health status profile of Rufiji district does not differ with the rest of the region, except for the health facilities in the delta zone, which were not included in our sample due to accessibility challenges. Lastly, study informants and participants were purposeful selected, and this implies that their viewpoints may not be representative of other individuals or settings.

## Conclusion

Our results suggest that the experience of unfairness in the way bonuses are distributed and administered on the health facility level undermines the legitimacy of the P4P scheme. Experiences and perceptions of health workers and their ideals of fairness varies across socio-cultural settings and yet important aspects to be considered during the design stages of P4P initiatives. Success or failure of such initiatives may depend to a significant degree on how P4P designs can balance the need for results, while maintaining and nurturing social relations in particular settings. This is a valuable insight considering that many P4P initiatives in many sub-Saharan African countries are either at pilot stage or in the process of being scaled-up.

## Policy implication and recommendations

In this section we will identify some policy implications and proffer a few policy recommendations based on our findings. Fairness principles vary across contexts but in this study it is demonstrated that effort has to be made to understand contextual perceptions and meanings of fairness. If this is not considered, a P4P scheme may lead to perceived unfairness, which negatively affects social relations among health workers and consequently service delivery. Broadening the scope of stakeholders that participate at the design stage of P4P programmes to including frontline health workers could help in capturing these contextual understandings of fairness and potentially avoid misunderstandings in P4P bonus allocations. Additionally, broadening the P4P indicators may also help in tackling the fairness issue. When indicators are wide enough, all health workers can contribute towards P4P targets and this may reduce conflicts at health facilities. In order to realize these recommendations, a bottom-up approach which actively involves all stakeholders including health workers and health service users is needed when considering P4P reforms in low-income context countries.

## Abbreviations

FGDs: Focus group discussions
IDIs: In-depth interviews
LICs: Low-income countries
MDGs: Millennium development goals
P4P: Pay-for-performance
RBF: Results-based financing
RCH: Reproductive and child health
